# Book Review: *Handbook of Embodied Psychology: Thinking, Feeling and Acting* by Michael D., Robinson, & Laura E., Thomas

**DOI:** 10.1177/03010066221109129

**Published:** 2022-07-06

**Authors:** Bettina Forster

**Affiliations:** School of Health and Psychological Sciences, Cognitive Neuroscience Research Unit, City, University of London, London, UK

Our brain is situated in a body that shapes the way the brain senses the environment around us, and it also shapes the way the brain acts upon the environment. In addition, the body itself and its inner states can serve to convey and process information. Thus, while psychology is the scientific study of brain and behaviour, embodied psychology considers the body when studying brain and behaviour. There are many examples that hint at a role of the body in our sensing of and acting upon the environment, and in mental processing and function, such as everyday expressions referring to ‘gut-feelings’ to explain a decision process (Huang & Pearce, 2015) or the influence of the position of our limbs on how we allocate visual attentional resources (Reed et al., 2006). Likewise, there are many theories that describe different ways the body is involved in perception, action and cognition. *The Handbook of Embodied Psychology: Thinking, Feeling and Acting* brings together different strands of empirical embodiment research from predominately the cognitive and social areas of psychology. It also features topics in neuroscience, personality and clinical psychology offering an overview of more recent developments. The empirical research reviews are framed by chapters on theoretical perspectives and chapters reflecting on future directions of embodiment research.

The first part of the book is a collection of chapters reviewing the main theoretical perspectives in embodied psychology which are later referred to throughout the empirical research reviews. These foundation accounts present theoretical perspectives on the grounding of concepts and cognition, bodily resource-based views of perception, interoception and metaphorical embodiment. An integrated account of these theories is not offered but it is acknowledged that a better understanding of the underlying mechanism of an embodiment is needed before these theories can be refined and, potentially, integrated. It is also pointed out that, at times, different theoretical accounts support different embodiment explanations, and more research is needed specifically in relation to such contrasting accounts. Yet, having such diverse embodiment theories in one place underlines the disparate theoretical embodied perspectives rooted in diverse areas of psychology and as such may stimulate future research and advances generating a (more) unified theoretical framework of embodied psychology.

The second and third parts of the book include a collection of reviews of empirical research first from cognitive and neuroscience perspectives, then from social and personality perspectives. The former part presents reviews that tackle embodiment in topics considering mathematical processing, abstract concepts, metacognition, language, attention, memory and virtual environments. The latter part presents reviews on the role of embodiment in topics including social cognition, affective synchrony, joint action, relationship dynamics, personality traits and clinical disorders. Each of the reviews is written by a different author or author group with their own emphasis on the topic at hand. This collection of reviews feels extensive and is relatively broad. Within the introduction brief summaries of the reviews are given by the editors and each of the review chapters starts with an abstract providing a useful gage of the content.

The final part of the book consists of five chapters on current issues and future directions. This collection of reviews and opinion pieces provides a broad context to the more empirical research within earlier chapters and points out crucial shortcomings of existing embodiment research. Some of these chapters are more reflective on current methodological approaches and others on the theoretical bases, giving pointers for future development needs in embodiment research. These and the introductory chapters within the first part are the most stimulating and thought provoking; while the 17 chapters within parts two and three each provide a solid overview of current empirical research within the different strands of cognitive and social embodiment research.

This book is a very well put together collection of reviews and opinion pieces on empirical research and theoretical approaches under the umbrella of embodied psychology. It is aimed at academics, researchers and graduate students interested in the wider field of embodied psychology. Several chapters deal with embodied perception (e.g. chapters 3, 5, 12 and 14) and may therefore be of particular interest to the readers of the journal *Perception.* Each chapter in this book is a stand-alone review that can be read in isolation. The introductory chapter by the editors provides a good overview of the book and a guide to select topics of interest. *The Handbook of Embodied Psychology: Thinking, Feeling and Acting* is a go-to reference book that I can imagine repeatedly coming back to in the future.
